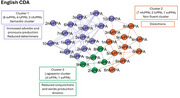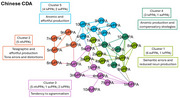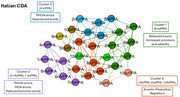# Cross‐linguistic characterization of speech and language profiles of English, Chinese, and Italian Primary Progressive Aphasia patients: a data‐driven approach

**DOI:** 10.1002/alz70857_106103

**Published:** 2025-12-24

**Authors:** Gaia Chiara Santi, Eleonora Catricalà, Stephanie Kwan, Anson Wong, Zoe Ezzes, Lisa D. Wauters, Valentina Esposito, Francesca Conca, Ta‐Fu Chen, Lorinda Li‐Ying Kwan Chen, Raymond Y. Lo, Joshua Tsoh, Lung‐Tat Chan, Adolfo M. Garcia, Jessica Deleon, Zachary Miller, Jet MJ Vonk, Isabel Elaine Allen, Elisa Canu, Federica Agosta, Massimo Filippi, Stefano F Cappa, Maria Luisa Gorno Tempini, Boon Lead Tee

**Affiliations:** ^1^ IRCCS Mondino Foundation, Pavia, Italy; ^2^ Institute for Advanced Studies, IUSS, Pavia, Italy; ^3^ Memory and Aging Center, Department of Neurology, University of California San Francisco, San Francisco, CA, USA; ^4^ The University of Texas at Austin, Austin, TX, USA; ^5^ Department of Neurology, National Taiwan University Hospital, Taipei, Taiwan; ^6^ Department of Special Education and Counselling, The Education University of Hong Kong, Hong Kong, Hong Kong; ^7^ Buddhist Tzu Chi General Hospital, Hualien, Taiwan; ^8^ Prince of Wales Hospital and ShaTin Hospital, Ma On Shan, New Territories, Hong Kong; ^9^ Department of Medicine, Queen Elizabeth Hospital, Hong Kong, Hong Kong; ^10^ Universidad Santiago de Chile, Estación Central, Santiago de Chile, Chile; ^11^ Global Brain Health Institute, University of California, San Francisco, CA, USA; ^12^ Cognitive Neuroscience Centre, University of San Andres, Victoria, Buenos Aires, Argentina; ^13^ Cognitive Neuroscience Center (CNC), Universidad de San Andres, Buenos Aires, CA, Argentina; ^14^ Department of Epidemiology and Biostatistics, University of California, San Francisco, San Francisco, CA, USA; ^15^ Neuroimaging Research Unit, Division of Neuroscience, IRCCS San Raffaele Scientific Institute, Milan, Italy; ^16^ Vita‐Salute San Raffaele University, Milan, MI, Italy; ^17^ Neurology Unit, IRCCS San Raffaele Scientific Institute, Milan, Italy

## Abstract

**Background:**

Speech and language profile of Primary Progressive Aphasia (PPA) has primarily been described based on English‐speaking cohorts. However, the potential influence of language typology in the clinical manifestation of PPA remains unclear. Using a data‐driven approach, we aim to examine the speech and language profiles of English, Chinese and Italian PPA and identify cross‐linguistic similarities and differences.

**Method:**

The study included 90 PPA (30 for each language, 10 for each variant). Speech samples, from the Picnic picture description, underwent CLAN analysis to characterize phonetic‐phonological, lexico‐semantic, morpho‐syntactic, and discourse‐pragmatic domains. Community detection analysis (CDA) was performed for each language separately, including speech, language, and demographic features. Emerged profiles were compared across languages.

**Result:**

English‐CDA resulted in 3 clusters (modularity:0.37; classified:100%). Cluster 1, including mainly svPPA, was characterized by reduced nouns production and compensatory strategies, including increased adverbs and pronouns production. Cluster 2, including mainly nfvPPA, was characterized by speech distortions. Cluster 3, predominated by lvPPA, presented with an anomic profile. Chinese‐CDA resulted in 5 clusters (Modularity:0.48; classified:100%). Cluster 1, including mainly svPPA, was characterized by reduced nouns production. Cluster 2 and 3 included mainly nfvPPA, with the former characterized by tone errors and distortions, the latter by a tendency to agrammatism. Cluster 4 and 5 included mainly lvPPA, with the former presenting anomic production and compensation strategies, the latter an anomic and reduced production. Italian‐CDA resulted in 4 main clusters (Modularity:0.34; classified:93%). Cluster 1, including mainly svPPA, was characterized by a reduced noun production and compensatory strategies. Cluster 2, including mainly nfvPPA, was characterized by phonological and morphological errors. Cluster 3 and 4 included mainly lvPPA, with the former presenting phonological disfunctions, the latter an increased words repetition.

**Conclusion:**

CDA results suggest a consistent semantic cluster across languages. Notably, nfvPPA and lvPPA patients displayed significant heterogeneity in their clustering and exhibited diverse speech and language features. These findings underscore the importance of language diversity in PPA research and highlight the role of language‐specific features in PPA clinical characterization (i.e., tone for Chinese, morphological errors for Italian). These findings advocate for the need for new diagnostic criteria in other languages.